# Top reviewers 2024 echo

**DOI:** 10.1111/echo.70123

**Published:** 2025-03-06

1


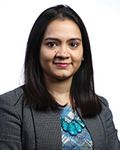


Dr. Ishita Datta:

**Dr. Ishita Datta** is a Pediatric Cardiology fellow at the Children's Hospital Los Angeles, Los Angeles, California. She has completed my medical training from Indira Gandhi Government Medical College, India followed by residency training in combined Internal Medicine and Pediatrics at Wayne State University, Detroit, Michigan. She has been passionate about research since her early days as a medical student and continue to find opportunities to contribute to research activities. In her career span so far, she has been served as a peer reviewer for several well established journals such as Echocardiography as well as for many national and international conferences.


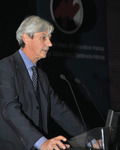


Dr. Cesare Cuspidi:

**Dr. Cesare Cuspidi** is Past Associate Professor of Internal Medicine at the University of MilanoBicocca, Medical School, Milan, Italy. He has authored of 560 full lengths papers, most of them in prestigious cardiovascular peerreviewed International journals. His research interests include hypertension and non‐invasive cardiology and he focused his attention several areas of clinical hypertension such as the assessment of cardiac and extra‐cardiac target organ damage, left ventricular hypertrophy regression, ambulatory blood pressure, cardiovascular risk stratification, metabolic /educational aspects and sleep apnoea syndrome. The total numbers of literature citations concerning his scientific production amount to 20094 and its HI index is 74. It was cited by the Expert Escape website based on 125,848 eligible articles published since 2013 among the 30 best hypertension experts worldwide.


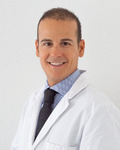


Dr. Dryfus Julien:

**Dr. Julien Dreyfus** is a cardiologist specializing in echocardiographic imaging, both in the lab and in interventional procedures. He is based at the Centre Cardiologique du Nord in Paris, France. His primary clinical and research focus revolves around Valvular Heart Disease, with a particular emphasis on tricuspid valve conditions, for which he earned his PhD.

He has held the position of President for the Young Cardiologists Group within the French Society of Cardiovascular Imaging and currently serves as a board member for several organizations, including the French Association of Valvular Heart Diseases, the French Society of Cardiovascular Imaging, the National College of French Cardiologists, and the Digital School of Cardiology. He also serves as Co‐Director for New Evidence in the PCR Tricuspid Focus Group, is an Associate Editor for JACC: Case Reports, and a member of the editorial board for the Echocardiography Journal of Cardiovascular Imaging and Intervention.


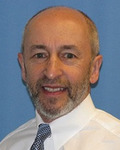


Dr. Charles Pollick:

**Dr. Charles Pollick** MBChB, FRCP, FASE, FACC is Director of Quality in the Echocardiography Laboratory, Faculty Staff Cardiologist, Associate Professor, Department of Medicine at Cedars‐Sinai Hospital and the Smidt Heart Institute in Los Angeles, and Health Sciences Assistant Clinical Professor Step III at David Geffen School of Medicine at UCLA. He has authored over 50 peer reviewed articles mainly related to hypertrophic cardiomyopathy and echocardiography which continue to be his main areas of research and interest.


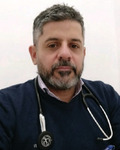


Dr. Adrian Carlessi:

**Dr. Carlos Adrián Carlessi** is an Argentine cardiologist specializing in cardiovascular imaging. He earned his medical degree from the Universidad Nacional de Rosario and completed his cardiology residency at ‘Dr. José María Cullen’ Hospital. With multiple master's degrees in advanced cardiology and cardiac imaging, he has held key roles in coronary care and echocardiography.

Dr. Carlessi is an active researcher, with numerous publications and presentations at international cardiology congresses, earning multiple awards. He also serves as a reviewer for the *Echocardiography Journal* and a speaker at cardiac imaging conferences.


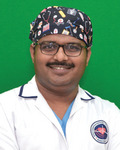


Dr. Maruti Haranal:

**Dr. Maruti Y. Haranal** is a highly skilled Cardiovascular and Thoracic Surgeon with over a decade of experience in pediatric and adult cardiothoracic procedures. He holds an MBBS from Shri B M Patil Medical College, an MS in General Surgery, and an MCh in Cardiothoracic and Vascular Surgery from Sri Jayadeva Institute of Cardiovascular Sciences. His advanced training includes fellowships at prestigious institutions like SickKids Hospital in Toronto and the National Heart Institute in Malaysia. Currently, he serves as a Consultant Pediatric Cardiac Surgeon at UN Mehta Hospital, India, specializing in complex congenital heart surgeries.

Dr. Haranal is an active researcher and has authored numerous publications in leading cardiothoracic journals. He has presented his work at international conferences and is a section editor for the *Indian Journal of Thoracic and Cardiovascular Surgery*. Recognized for his contributions, he has received multiple awards and remains committed to advancing cardiac surgery through research, innovation, and mentoring future surgeons.


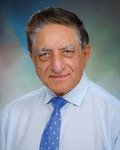


Dr. Ahmad Masood:

**Dr. Masood Ahmad** is a distinguished cardiologist and Professor of Medicine, serving as the Director of Echocardiography at the University of Texas Medical Branch (UTMB). With over four decades of experience, he has been instrumental in advancing echocardiography, overseeing the growth of UTMB's lab from 1,200 to over 16,000 procedures annually. His expertise in 3D and contrast echocardiography has positioned UTMB as a leader in the field. Dr. Ahmad has trained countless sonographers, residents, and fellows, maintaining a 100% board pass rate for his trainees. He also plays a pivotal role in clinical care, research, and education, contributing significantly to patient care innovations and the development of echocardiographic techniques.

A respected researcher, Dr. Ahmad has led multiple clinical trials and published extensively in peer‐reviewed journals. He serves as an Associate Editor for the *Journal of Echocardiography* and holds leadership roles in professional societies, including the International Society of Cardiovascular Ultrasound. His contributions to noninvasive cardiovascular imaging have earned him numerous accolades, including the Edward D. and Sally M. Futch Endowed Professorship in Cardiology. Recently appointed Professor Emeritus, he continues to influence the field through mentorship, research, and clinical excellence.


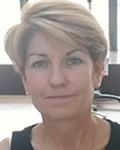


Dr. Floria Mariana:

Professor at “Grigore T. Popa” University of Medicine and Pharmacy; senior consultant in internal medicine and cardiologist at Emergency Clinical Hospital Iași, Romania; Fellowship in arrhythmology (two years) and echocardiography (two years) at Catolic University of Louvain, in Belgium ; EACVI certification in transthoracic echocardiography ; Fellow of European Society of Cardiology; Hirsh index: 16 ; https://orcid.org/0000‐0002‐9465‐1503.

I have a particular interest in cardiac rhythm disorders (especially atrial fibrillation) and cardiovascular imaging (especially echocardiography). My research work is related to atrial fibrillation and other arrhythmias in patients with cardiac comorbidities.


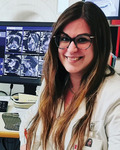


Dr. Anna Pavon:

**Dr. Anna Giulia Pavon** is a multimodality cardiac imager, skilled in clinical cardiology and imaging techniques including transthoracic and transesophageal echocardiography, cardiac magnetic resonance imaging, and cardiac computed tomography. Dr. Pavon graduated in Medicine at Vita‐Salute San Raffaele University in Milan, Italy where she also completed the residency program in Cardiovascular Disease. She worked for several years as Cardiovascular Magnetic Resonance expert in the “Centre pour la Résonance Magnétique Cardiaque” in Lausanne (Switzerland) and as consultant cardiologist in the Cardiology Department of the Centre Hospitalier Universitaire Vaudoise, Lausanne, Switzerland.

Since 2021 she has been appointed as senior cardiologist in cardiovascular imaging at “Istituto Cardiocentro Ticino”, Lugano, Switzerland. Member of different working group and committee for both the European Association of Cardiovascular Imaging (EACVI) and for the Society of Cardiovascular Magnetic Resonance (SCMR) she has published several papers on cardiovascular imaging in high rank journals and participated as speakers in national and international meetings.


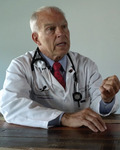


Dr. Robert Gatewood:

After graduating from Georgetown Medical School in 1974 and completing an Internal Medicine residency at SUNY Buffalo, Dr. Robert P. Gatewood, Jr. began his cardiology training at the University of Rochester Strong Memorial Hospital. During his 3 years in Rochester, he developed a close relationship with Dr. Navin Nanda who has been his inspiration and mentor over the past 54 years. It was under his tutelage that Dr. Gatewood passionately embraced echocardiography. During his career practicing noninvasive clinical cardiology, he has had the opportunity to speak both nationally and internationally on various subjects in the field of echocardiography. As one of the original partners of Buffalo Cardiology and Pulmonary Associates in Buffalo New York, he would routinely be the first to introduce new advances in echocardiography to the Buffalo area, from 2D echo to 3D echo, conventional Doppler to color Doppler, single plane to multiplane transesophageal echocardiography (TEE), and point‐of‐care ultrasound (POCUS) to intraoperative echocardiography. His vast clinical experience and his research efforts with Dr. Nanda have been the major underpinnings of his broad knowledge of echocardiography. In addition to his work in echocardiography, he serves on the faculty at the Jacobs School of Medicine and Biomedical Sciences at University of Buffalo.

